# Development of a method based on ultra high performance liquid chromatography coupled with quadrupole time-of-flight mass spectrometry for studying the in vitro metabolism of phosphorothioate oligonucleotides

**DOI:** 10.1007/s00216-015-9266-1

**Published:** 2016-01-12

**Authors:** Sylwia Studzińska, Rafał Rola, Bogusław Buszewski

**Affiliations:** Chair of Environmental Chemistry and Bioanalytics, Faculty of Chemistry, Nicolaus Copernicus University, 7 Gagarin St., PL-87 100 Torun, Poland

**Keywords:** Phosphorothioate oligonucleotides, Ion pair chromatography, Separation, Sample preparation, In vitro metabolism

## Abstract

Ultra high performance liquid chromatography hyphenated with quadrupole time-of-flight mass spectrometry was used to determine the products of the in vitro metabolism of phosphorothioate oligonucleotides. These compounds may be used during antisense therapy as synthetic fragments of genes. For this reason, both a sample preparation method and a qualification method were developed during this study. Liquid–liquid extraction, protein or oligonucleotide precipitation, and solid-phase extraction were tested and compared in order to select the method that yielded the highest recoveries. Ion pair chromatography was used for separation while mass spectrometry was applied for metabolite identification. The influence of the type of ion pair reagent used on the resolution and sensitivity was investigated. Results indicated that a mixture of 1,1,1,3,3,3-hexafluoro-2-propanol, *N*,*N*-dimethylbutylamine, and methanol was the best mobile phase for maximizing both of these parameters. The developed method was applied to investigate the compounds that form during the incubation of phosphorothioate oligonucleotides with human liver microsomes. Metabolites with short sequences were created after 8 hours, while oligonucleotides constructed from a large number of nucleotide units were obtained after 12 hours of incubation. Moreover, regardless of the length of the polynucleotide chain, metabolites were produced by the same mechanism: enzymatic cleavage at the 3′ end of the sequence.

## Introduction

Antisense oligonucleotides (ASOs) are single-stranded modified fragments of both deoxyribonucleic acid (DNA) and ribonucleic acid (RNA) of exogenous origin [1, 2]. They typically consist of 12–20 nucleotide units. These compounds are capable of binding to a specific fragment of DNA, RNA, and even proteins [2]. The sequence of nitrogenous bases present in ASO is complementary to the sequence of a forward DNA or mRNA [1, 2]. ASOs may take part in the inhibition of transcription and translation processes, which makes them an efficient tool for inhibiting the expression of a particular gene [3]. The structure of an ASO needs to be chemically modified with various functional groups such as sulfur, methyl, methoxy, or fluorine moieties [3, 4] in order to increase its stability to enzyme attack and to increase the hydrophobicity of the oligonucleotides so that penetration through biological membranes is facilitated [4].

Because of their ability to inhibit gene expression, ASOs are used and tested in treatments for many diseases. Over 40 of them are currently at various stages of clinical trials, and three have already been launched onto the pharmaceutical market [4–7]. ASOs show therapeutic effects in treatments for diseases of the cardiovascular system (e.g., atherosclerosis), breathing problems (e.g., asthma), inflammation, infectious diseases (e.g., HIV, influenza), and especially in tumors [5, 8]. Nearly half of the ASO-based drugs that are undergoing clinical trials are being investigated for their anticancer therapy potential [5, 8]. Researchers working with ASOs continue to face numerous challenges, such as ensuring their specificity of action, stopping the ASOs from simultaneously inhibiting the expression of several genes, identifying appropriate drug-delivery methods, and—most importantly—determining possible products of the metabolism of ASOs in the human body [5, 9].

The metabolism model of ASO assumes that there is considerable exonuclease activity leading to nucleotide cleavage at the ends of the polynucleotide chain, with a much smaller level of endonuclease activity leading to the hydrolysis of intrachain phosphodiester bonds [10]. It has been shown that the greater contribution of exonucleases to the degradation of ASO is linked to the activity of the 3′ end as compared to that of the 5′ end [11]. Crooke et al. [11], based on studies performed using rat liver homogenate, proved that nuclease activity is affected by the concentration of the ASO, pH, and ionic strength. Moreover, sequences with a relatively high proportion of pyrimidine bases (cytosine, thymine) show faster enzymatic digestion than oligonucleotides of the same length with a high proportion of AG/CT [11]. Furthermore, it was also shown in the literature that oxidation is not involved in the metabolism of ASOs [11, 12].

Qualitative and quantitative analyses of active metabolites are crucial because they provide valuable information about the activity and potential side effects of the metabolites. Moreover, they lead to a greater understanding of the complexities involved in the metabolism of ASOs. Ion-pair reversed-phase high-performance liquid chromatography (IP RP HPLC) coupled with mass spectrometry (MS) is the method that is most commonly used to separate and to quantitatively and qualitatively analyze metabolites [11–14].

This technique was successfully used to analyze the products of the metabolism of phosphorothioate oligonucleotide incubated in a solution of 3′-exonuclease [14]. However, the use of IP RP HPLC coupled with tandem mass spectrometry (MS/MS) permitted the separation and identification of ASO metabolites produced in vivo, e.g., in rat plasma [14, 15]. Dai et al. [16] analyzed the metabolism of biomolecules by examining the plasma samples of patients treated with antisense therapy. This characterization of metabolites was possible due to the ability to perform very accurate molecular weight measurements (±0.009 %) using Q-TOF MS [16]. During in vitro and in vivo metabolism studies performed using a variety of biological samples (aqueous solution of exonucleases, plasma, urine, homogenate of mouse liver/kidney), 3′ N-shorter oligonucleotides were detected [17, 18]. Only one type of stationary phase was used for UHPLC in all of these studies, namely octadecyl packing material. There are no studies concerning the application of other columns to separate these metabolites. Moreover, no systematic studies on complex method development have been published to date. Therefore, our goal was to investigate, in a systematic way, the influences of various method parameters (stationary phase type, mobile phase composition, MS parameters, sample preparation method employed, various parameters associated with microsomal incubation) on separation efficiency and sensitivity.

Consequently, the main aim of the study reported in the present paper was to investigate the in vitro metabolism of phosphorothioate oligonucleotides. Systematic studies on analytical method development were performed. IP RP HPLC coupled with Q-TOF MS was utilized during the research in order to separate metabolites and identify them. The influences of the type and concentration of the ion-pair reagent used on the retention of oligonucleotides and the MS detection sensitivity were tested. Moreover, various sample preparation techniques were investigated to identify the method that yielded the highest recoveries. The developed methods were successfully used during metabolism studies employing human liver microsomes.

## Materials and methods

### Oligonucleotide samples and chemicals

Phosphorothioate standards of oligonucleotides were purchased from Sigma–Aldrich (Poole, UK). The sequences of the analyzed compounds together with their masses are presented in Table 1. They were supplied in lyophilized form and standard solutions were prepared by dissolution in deionized water. The concentration of each stock solution was 0.1 mM.

**Table 1:** Properties of the oligonucleotides used in this study

Name	Molecular mass (g mol^−1^)	Boiling point (°C)	Sequence (5′–3′)
OBL	5683.7436	72.9	TCTCCCAGCGTGCGCCAT
TRA	6367.1245	74.9	GCCCAAGCTGGCATCCGTCA
ISI21	6349.8581	72.1	TCCGTCATCGCTCCTCAGGG
ISI20	6434.5587	63.1	GTTCTCGCTGGTGAGTTTCA
ISI19	6105.5042	60.0	GTTCTCGCTGGTGAGTTTC
ISI18	5799.2314	57.9	GTTCTCGCTGGTGAGTTT
ISI17	5479.3652	56.1	GTTCTCGCTGGTGAGTT
ISI16	5159.7771	54.0	GTTCTCGCTGGTGAGT
ISIA18	5768.6956	54.4	CGGCATGTCTATTTTGTA
ISIA17	5437.4883	54.1	CGGCATGTCTATTTTGT
ISIA16	5118.1224	52.8	CGGCATGTCTATTTTG
ISIA15	4773.3569	48.9	CGGCATGTCTATTTT
ISIA14	4452.4288	23.0	CGGCATGTCTATTT

Mobile phases were prepared for mass spectrometry, using organic solvents such as methanol and 1,1,1,3,3,3-hexafluoro-2-propanol (HFIP) (Sigma-Aldrich, Dorset, UK). These mobile phases also contained ion-pair reagents: triethylamine (TEA), *N*,*N*-dimethylbutylamine (DMBA), and hexylamine (HA), which were purchased from Sigma–Aldrich. Deionized water was obtained from a Milli-Q system (Millipore, El Passo, TX, USA).

The following solvents were used during sample preparation: chloroform, sodium dodecyl sulfate (SDS), isopropanol, sodium tris-EDTA, hydrochloric acid, and phenol (POCh, Gliwice, Poland). Nicotinamide adenine dinucleotide phosphate (NADP), dehydrogenase, glucose-6-phosphate, sodium phosphate (Na_2_HPO_4_), sodium dihydrogen phosphate dihydrate (NaH_2_PO_4_⋅H_2_O), and microsomes derived from human liver cells (HLM) at a concentration of 10 mg/vial were used in the enzymatic studies. All of these reagents were also purchased from Sigma–Aldrich.

### Instrumentation

A 1260 Infinity quaternary ultra high performance liquid chromatography (UHPLC) system (Agilent, Waldbronn, Germany) equipped with a binary pump, vacuum-chambered microdegasser, thermostatically controlled autosampler, and column compartment were used in the study. The system was also equipped with a diode-array detector and an Agilent 6540 UHD accurate-mass quadrupole time-of-flight (Q-TOF) mass spectrometer. Electrospray ionization (ESI) was applied in negative ion mode. A full-scan MS was recorded within the mass range *m*/*z* 400–1650 and then quadrupole precursor ion selection MS/MS was realized for *m*/*z* 50–1650. The following MS/MS parameters were applied: drying gas flow rate 11.0 L min^−1^; shielding gas flow rate 10.0 L min^−1^; nebulizer gas pressure 20 psi; skimmer voltage 60 V, octopole voltage 750 V; capillary voltage 4000 V; shielding/drying gas at 350/400 °C; fragmentor voltage 150 V. Nitrogen was used in the ion source and the collision cell. Data were collected using the Agilent Mass Hunter software, version B.04.01.

Sample preparation was performed using a CentriVap vacuum concentrator (Labconco, Kansas City, MO, USA), a model 5424 centrifuge (Eppendorf AG, Hamburg, Germany), a ThermoMixer (VWR, Randor, PA, USA), a laboratory pH meter (Elmetron CP-505, Zabrze, Poland), and Oasis^®^ HLB columns for solid-phase extraction (60 mg/3 ml; Waters, Milford, MA, USA).

### Chromatographic analysis

Chromatographic analyses were performed with a Hypersil GOLD C18 selectivity LC column (1.9 μm particle size; 2.1 × 100 mm; Thermo Fisher Scientific, Waltham, CA, USA). The mobile phase flow rate was equal to 0.3 mL min^−1^. The autosampler temperature was 30 °C, while the column was kept at 50 °C. The injection volume was 1 μL. The column void volume (*t*_0_) was measured by methanol injection. The UV–vis detection wavelength was selected as *λ* = 260 nm.

The retention studies were carried out in the isocratic elution mode in order to investigate the influence of ion-pair reagent structure on the retention of oligonucleotides. The separation of metabolites was carried out using an appropriate gradient to reduce the time needed for analysis. The mobile phase consisted of methanol, 150 mM HFIP, and 5 mM TEA or DMBA or HA in water. Various levels of methanol were applied to the mobile phase, depending on the concentration of the ion-pair reagent. However, the mobile phase that was ultimately chosen consisted of 150 mM HFIP, 5 mM DMBA, and methanol.

### Incubation with HLM enzymes

The incubation of phosphorothioate oligonucleotide with HLM was performed in 100 mM phosphate buffer (pH 7.4) at 37 °C. The incubated samples were shaken for a specified period of time at 300 rpm. Optimization of the conditions employed to carry out the process included the incubation time, the concentration of HLM, the concentration of analytes, and the reaction buffer (consisting of the enzymatic reaction cofactor NADP/NADPH, glucose-6-phosphate (G-6-P) dehydrogenase, and magnesium ions). Blank samples had the same compositions as the corresponding test samples but did not contain oligonucleotide; phosphate buffer was added instead in order to maintain a constant total sample volume. The process was terminated with 50 μl of ice-cold ACN. Another sample was immediately prepared and analyzed via UHPLC Q-TOF MS. All samples were prepared in triplicate. The parameters selected for final incubation were as follows: 12 h of incubation time, 1 mg mL^−1^ of HLM, 30 μM of ISIA18 and ISI20, 3 mM of NADP/NADPH, 20 mg mL^−1^ G-6-P, 20 U mL^−1^ of dehydrogenase, and 10 mM of MgCl_2_.

### Sample preparation

Several different sample preparation methods were tested during the present investigation: protein precipitation with acetonitrile, liquid–liquid extraction with phenol/chloroform, solid-phase extraction, and denaturation of proteins by 20 % SDS solution and ammonium acetate (followed by precipitation of oligonucleotides using isopropanol or ethanol).

During the extraction with a phenol/chloroform mixture, appropriately prepared basic phenol was used. This involved melted the phenol at a temperature >68 °C, adding a double volume of 0.4 M Tris–HCl (pH 8.0), stirring, and, after phase separation, removing the aqueous layer using a pipette. The solution was washed with Tris–HCl until the pH of the phenol was >7.8. A significant proportion of the isolated DNA remained in the organic phase at lower pH values.

## Results and discussion

### Influence of the ion-pair reagent on the retention and separation of oligonucleotides

IP RP HPLC was selected for the development of a chromatographic method of separating ASOs and their metabolites, due to their polarity. Since MS detection was used, volatile mobile phases were chosen. HFIP was utilized, as it is has a lower boiling point than acetic acid. Furthermore, amines are less soluble in HFIP [19], so they are more effectively adsorbed on the surface of the stationary phase than acetates are [19]. Three different amines were used during the investigations: triethylamine, *N*,*N*-dimethylbutylamine, and hexylamine. All of these have similar emprical formulae although they differ structurally. The concentration of HFIP was 150 mM while that of the amine was 5 mM. Methanol was used as an organic solvent because of its good solubility in HFIP.

Table 2 presents the retention factors (*k*) of the analyzed compounds obtained during the study. In the case of the mobile phase consisting of HFIP/TEA, 18 % v/v methanol was used. When the same percentage part of methanol was added to mobile phases containing DMBA or HA, the *k* values were greater than 60. Instead, comparable *k* values to that obtained with TEA were gained by using 25 % v/v MeOH with DMBA and 47 % v/v with HA. This phenomenon is due to the structures of the amines used in the retention studies. The linear alkyl chain with six carbon atoms in HA leads to stronger hydrophobic chain–chain interactions between the amine and the octadecyl ligands on the stationary phase surface than when TEA or DMBA are used. The strength of these interactions affects the extent to which the HA is adsorbed on the packing material. Consequently, it also influences the number of dynamically formed anion-exchange centers on the surface of the stationary phase. Therefore, there are stronger electrostatic interactions in the mechanism of ASO retention when HA is a component of the chromatographic solvent.

**Table 2:** Retention factor (*k*) values determined for the studied oligonucleotides and for different types of ion-pair reagents

Compound	*k*		
	HFIP/TEA	HFIP/DMBA	HFIP/HA
	(18 % v/v MeOH)	(25 % v/v MeOH)	(47 % v/v MeOH)
ISIA18	2.10	1.59	2.29
ISI21	2.90	2.04	4.83
ISI20	3.27	2.43	5.28
OBL	1.24	0.94	2.10
TRA	2.27	1.70	3.68

It was decided that TEA should be excluded from subsequent studies for a variety of reasons: the low *k* values determined for this amine, the low percentage of methanol in the mobile phase with TEA, and, more importantly, the so-called memory effect caused by TEA during MS detection. This amine is usually produces an intense signal in mass spectra that suppresses signals from other analytes and increases the detection limit [20].

**Fig. 1a–d:**
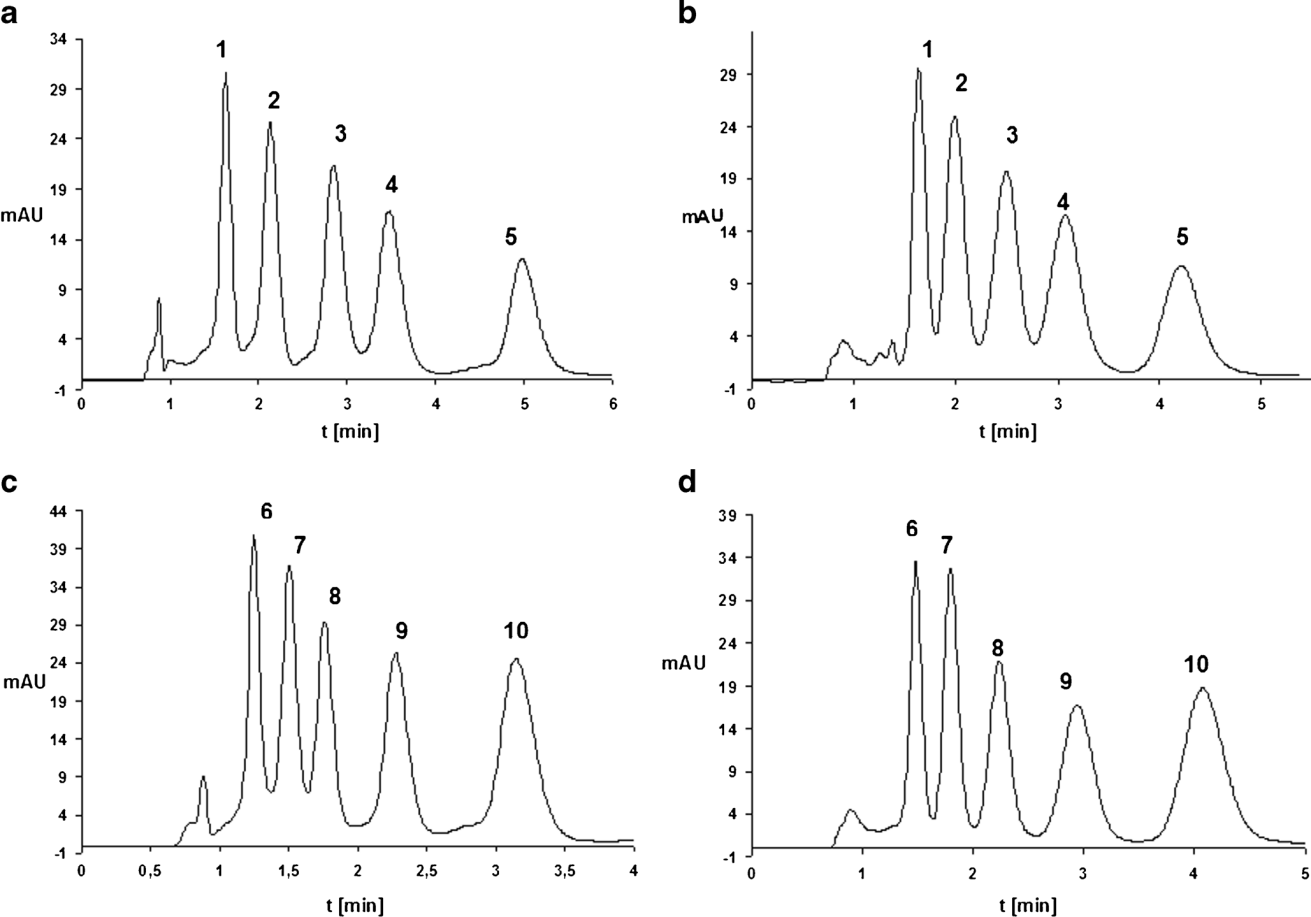
Chromatograms for the separation of ISI20 or ISIA18 and their synthetic metabolites: **a** ISI20 and its metabolites, mobile phase composition: 150 mM HFIP/5 mM DMBA and MeOH, gradient elution from 24.5 % to 28.5 % v/v MeOH in 5 min; **b** ISI20 and its metabolites, mobile phase composition: 150 mM HFIP/5 mM HA and MeOH, gradient elution from 47.5 % to 52 % v/v MeOH in 6 min; **c** ISIA18 and its metabolites, mobile phase composition: 150 mM HFIP/5 mM DMBA and MeOH, gradient elution from 24.5 % to 28.5 % v/v MeOH in 5 min; **d** ISIA18 and its metabolites, mobile phase composition: 150 mM HFIP/5 mM HA and MeOH, gradient elution from 46.5 % to 50 % v/v MeOH in 5 min. Chromatographic column was a Hypersil Gold C18, UV detection at *λ *= 260 nm; column temperature 50 °C, autosampler temperature 40 °C, flow rate 0.3 ml/min. Notation: *1* ISI16, *2* ISI17, *3* ISI18, *4* ISI19, *5* ISI20, *6* ISIA14, *7* ISIA15, *8* ISIA16, *9* ISIA17, *10* ISIA18

The influence of the type of amine used in the mobile phase on the separation of the ASO mixture was studied in the next set of investigations. The mixtures analyzed contained the parent compounds and their synthetic metabolites, which were formed by removing consecutive nucleotides from both the 3′ and 5′ ends of the sequence. Examples of these chromatograms are shown in Fig. 1. The highest values of *k* were obtained for the parent oligonucleotide, as it has the largest number of nucleotides in its sequence (Table 1). The longer the polynucleotide chain, the greater the retention of the studied biomolecule. The order of elution of the phosphorothioate oligonucleotides did not depend on the type of stationary phase or ion-pair reagent used. Based on the chromatograms shown in Fig. 1, it is evident that DMBA and HA can be used interchangeably as the ion-pair reagent, since both permit the complete separation of ASOs and their metabolites in a short period of time (Fig. 1). The utilization of UHPLC in the analysis of phosphorothioate oligonucleotides allowed us to reduce the amount of sample and the volumes of solvents required, to shorten the analysis time, and to increase the separation efficiency. This technique appears to be crucial to the successful analysis of ASOs.

### Optimization of the parameters associated with the analysis of oligonucleotides by mass spectrometry

The following parameters associated with the use of mass spectrometry in the analysis of phosphorothioate oligonucleotides were optimized: temperature of the drying/shielding gas, pressure in the nebulizer, capillary voltage, and the fragmentor, skimmer, and octopole voltages.

The associated analyses were performed in negative ion mode with the use of electrospray ionization (ESI). Four compounds were selected for this purpose: ISIA18 and its metabolite ISIA15, as well as ISI20 and its metabolite ISI17 (Table 1). The peak area in the extracted ion chromatogram (EIC) for the range *m*/*z* = 400–2500 Da was the parameter compared. Tests were conducted for two different mobile phases, one containing HA and the other DMBA. However, similar results were observed for both amines.

In the first stage, the drying gas temperature was varied in the range 250–300 °C, the shielding gas was varied in the range 300–400 °C; nebulizer pressure was varied in the range 20–50 psi; capillary voltage was varied in the range 3000–4000 V; fragmentor voltage was varied in the range 15–250 V; skimmer voltage was varied in the range 10–110 V; and octopole voltage was varied in the range 550–800 V. The peak area in the EIC was found to be maximized for a shielding/drying gas temperature of 350/400 °C, a nebulizer pressure of 20 psi, a capillary voltage of 4000 V, a fragmentor voltage of 150 V, a skimmer voltage of 60 V, and an octopole voltage of 750 V. These results were similar for both mobile phases (150 mM HFIP/5 mM DMBA/MeOH and 150 mM HFIP/5 mM HA/MeOH). Consequently, these parameter values were used during the present investigation for MS detection.

However, different results were observed when comparing the EIC peak areas obtained using the optimized MS parameters and mobile phases composed of DMBA, HA, or mixtures of them with HFIP and methanol. When the former amine was used, the peak areas were four times higher than those obtained with HA. Consequently, the MS sensitivity was greater when DMBA was used. Indeed, DMBA was found to give larger peak areas than HA regardless of the concentration of the amine ion-pair reagent used. Thus, mobile phases composed of methanol, HFIP, and DMBA were used in subsequent analyses.

**Fig. 2a–d:**
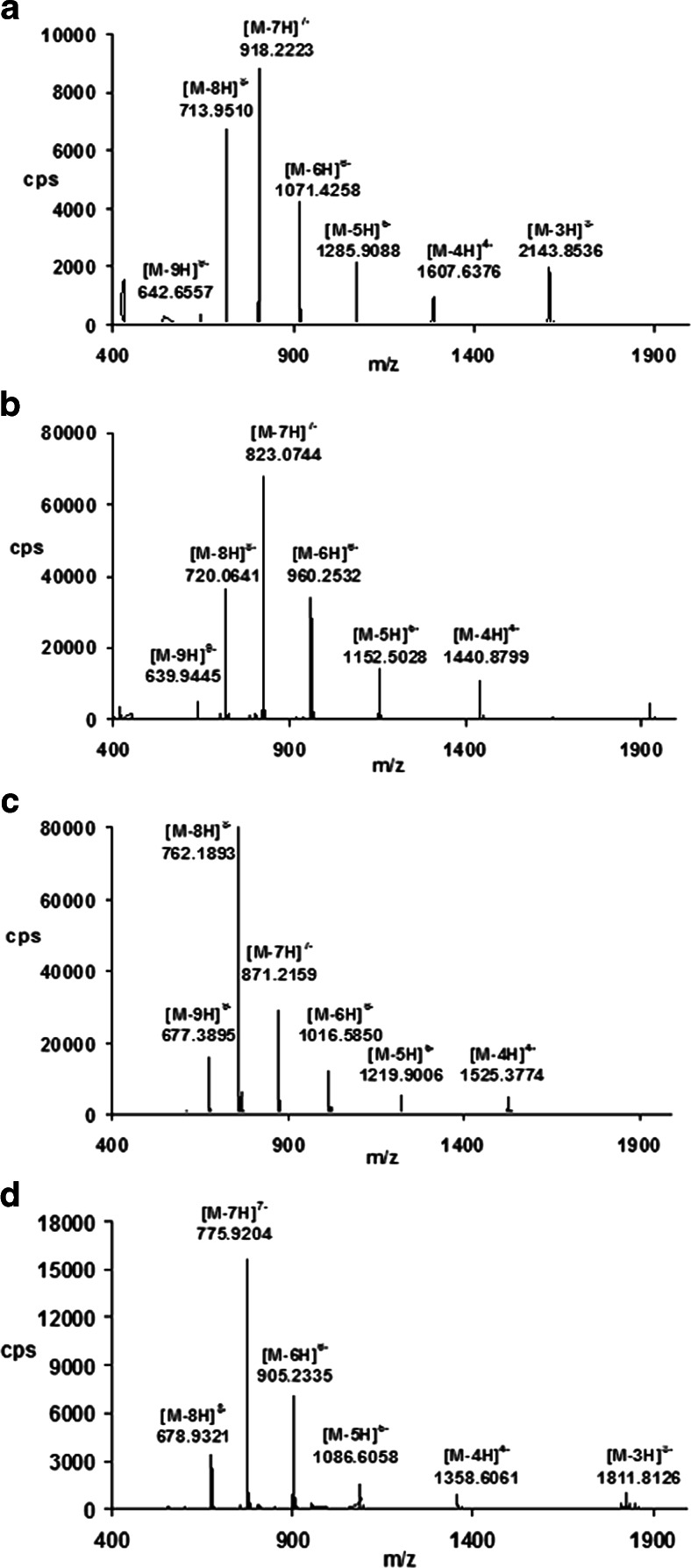
Mass spectra of phosphorothioate oligonucleotides: **a** ISI20, **b** ISIA18, **c** ISI19, **d** ISIA17. Analytical conditions: chromatographic column, Hypersil Gold C18; mobile-phase composition, 75 % v/v 150 mM HFIP/5 mM DMBA and 25 % v/v MeOH; column temperature, 50 °C; autosampler temperature, 40 °C; flow rate, 0.3 ml/min; drying/shielding gas temperature, 350/400 °C; nebulizer gas pressure, 20 psi; skimmer voltage, 60 V, octopole voltage, 750 V; capillary voltage, 4000 V; fragmentor voltage, 150 V

Examples of the full-scan mass spectra obtained for phosphorothioate oligonucleotides are shown in Fig. 2. A series of peaks can be seen, which are derived from multiply charged ions (M-*n*H)^*n*−^ due to the polyanionic character of the studied biomolecules. Each signal corresponds to an oligonucleotide that has lost some protons from the phosphorothioate group. Deconvolution was used to determine the molecular masses of the analyzed biomolecules. The mass error was equal to ±0.0007 Da. Moreover, the sensitivity of MS was determined based on the EIC peak area (the parent ion was the most intense signal in the full-scan spectra). The limit of quantification was in the range 1.0–1.3 μg mL^−1^.

The spectra were recorded in the range *m*/*z* 400–2500 Da. No oligonucleotide signals were observed below 400 Da, while signals from HFIP and its dimer were present.

### Product ion mode in the analysis of phosphorothioate oligonucleotides

In the next phase of our investigation, MS/MS was used to determine whether the fragmentation of oligonucleotides was specific and allowed for their rapid identification in subsequent steps, e.g., in the determination of metabolites. The most intense signal in the full-scan spectra was chosen as the parent ion.

We first focused on optimizing the collision energy. Energies ranging from 10 to 200 eV in steps of 10 eV were tested. The greatest number of fragment ions was obtained for 40 eV, so this collision energy was selected for further studies.

**Fig. 3a–d:**
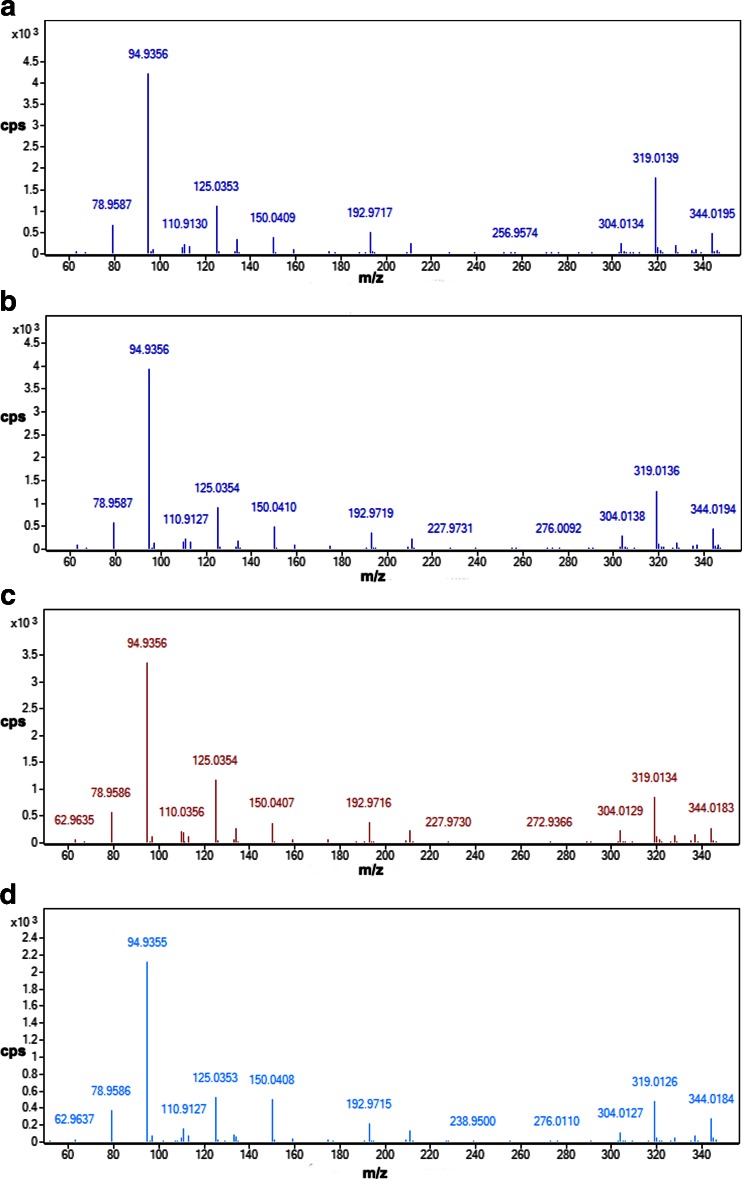
Product ion spectra for **a** ISIA18, **b** ISI20, **c** ISIA14, and **d** ISI16. Analytical conditions: chromatographic column, Hypersil Gold C18; mobile phase composition, 75 % v/v 150 mM HFIP/5 mM DMBA and 25 % v/v MeOH; column temperature, 50 °C; autosampler temperature, 40 °C; flow rate, 0.3 ml/min; drying/shielding gas temperature, 350/400 °C; nebulizer gas pressure, 20 psi; skimmer voltage, 60 V, octopole voltage, 750 V; capillary voltage, 4000 V; fragmentor voltage, 150 V; collision energy, 40 eV

All of the studied ASOs yielded identical fragmentation spectra (see Fig. 3). Regardless of the sequence of the ASO and its content of individual bases, identical signals from the structural elements of the oligonucleotides were observed in the spectrum. These signals came from phosphorothioate groups, nitrogenous bases, or nucleotides. During the fragmentation of the test biomolecules, water molecules dissociate from the sugar unit at the 5′ or 3′ end. The most intense fragment ion occurred at 94.9 Da; this was derived from a phosphorothioate group (characteristic of the studied compounds). Signals at *m*/*z* values such as 110, 125, 134, and 150 Da corresponded to the four nitrogen bases from which the oligonucleotides were constructed. Signals at *m*/*z* 304, 319, and 344 Da were related to nucleotides. In addition, a fragment ion corresponding to deoxyribose (*m*/*z* 193 Da) was also observed.

The results obtained showed that MS/MS would not be useful as a tool for qualitative analysis because it is not possible to confirm the structures of the metabolites based on the product ion spectra.

### Sample preparation for the identification of phosphorothioate oligonucleotides

A number of different methods of preparing samples for chromatographic analysis were tested in the course of the present investigation. The first was precipitation with acetonitrile. After fortifying the sample with ASO, it was centrifuged and the supernatant was analyzed by UHPLC in ion-pair mode. However, none of the studied biomolecules were determined in the sample using this method, due to the co-precipitation of the oligonucleotides with proteins (oligonucleotides have a high affinity for proteins).

The standard method for isolating genetic material was used in the next step of the study: liquid–liquid extraction with a phenol/chloroform (1:1) mixture was applied. These solvents were added to a sample fortified with oligonucleotides. The resulting mixture was shaken and centrifuged (13,000 rpm for 30 min). Denatured proteins were found at the boundary between the aqueous and organic phase, while ASOs were observed to be present in the upper aqueous phase. However, due to the solubility of phenol in water, there was a high concentration of this solvent in the supernatant, together with the oligonucleotides. The signal from phenol suppressed the signals of the studied molecules, ruling out MS detection as a viable option. The residue of phenol was successfully removed from the sample by fivefold liquid–liquid extraction with chloroform in the ratio 1:4. The recovery of ASO from the sample was 95 %.

Another sample preparation method that was tested was based on the denaturation of proteins by 20 % SDS solution and ammonium acetate, followed by the precipitation of oligonucleotides using ethanol. After evaporating the alcohol, the precipitated compounds were dissolved in the mobile phase. Chromatographic analysis of the resulting solution revealed that the recovery of oligonucleotides was 16 %. This is a consequence of the small size of the oligonucleotides of interest, which are sequences of only 21 nucleotides, whereas this method is more suitable for nucleic acids comprising several thousand nucleotides.

Solid-phase extraction (SPE) was also utilized for the extraction, concentration, and purification of the studied ASOs from incubated samples. SPE was performed in several steps using Oasis^®^ HLB SPE columns, which are commonly used to isolate oligonucleotides [17, 18]. The SPE adsorbent was conditioned with methanol and a buffer consisting of 400 mM HFIP and 15 mM DMBA. Higher concentrations of HFIP and DMBA were used here than in the mobile phase employed in the IP RP HPLC due to expected surface saturation with the ion-pairing reagent. The sample was then loaded onto the sorbent and the system was left for 30 min to allow oligonucleotides to be adsorbed through ion-pair formation. Different concentrations of HFIP and DMBA were used in the washing and eluting solutions, and different proportions of organic solvent were applied. The highest recovery was obtained for 150 mM HFIP and 5 mM DMBA. Next, the SPE column was washed first with water and then with a solution of 90 % v/v 150 mM HFIP/5 mM DMBA and 10 % v/v of methanol.in order to remove unbound substances from the surface of the adsorbent. Phosphorothioate oligonucleotides were eluted with mixture of 50 % v/v 150 mM HFIP/5 mM DMBA and 50 % v/v of methanol. Gravity flow was used at all stages of the SPE procedure. The prepared sample was subsequently transferred to a vacuum concentrator to evaporate the solvent at 65 °C. The residue was dissolved in 20 μl of mobile phase and analyzed with UHPLC. Some of the SPE parameters (time permitted for the adsorption of oligonucleotides on the surface of the SPE column; the volumes of the solvents used for conditioning, washing, and leaching) were varied to determine the effects of these parameters on the process of extraction. The results of the chromatographic analysis proved that changing these parameters did not significantly influence the final result. Phosphorothioate oligonucleotide recovery from the incubation samples was in the range 21–25 %. The application of SPE led to the effective separation of oligonucleotides from other components of the matrix. However, the low recoveries obtained prevented this technique from being used subsequently in this study.

Based on the results obtained, phenol/chloroform extraction was selected as the preparation method for subsequent investigations, due to its brevity, the high recoveries obtained, its low cost, and its effective removal of contaminants.

### In vitro incubation of oligonucleotides with human liver microsomes

The main organ in which oligonucleotide biotransformation occurs is the liver. In vitro studies must accurately reflect the phenomena that occur in the human body if the results are to be used in the development of clinical applications. Microsomes are commonly used in these systems so they were also utilized during the present investigation. Two phosphorothioate oligonucleotides were included in the incubation tests: ISIA18 and ISI20 (Table 1).

Incubation of the microsomal fraction with enzymes was performed in phosphate buffer (pH 7.4) at 37 °C. The incubated samples were shaken for a specified length of time at 300 rpm. The values of several parameters associated with this process were varied in order to optimize them; these included the incubation time (2–24 h), the concentration of HLM (0.25–2 mg mL^−1^), the concentration of oligonucleotides (20–30 μM), and the concentrations of the components of a reaction buffer consisting of the enzyme reaction cofactor NADP/NADPH (1.29–20 mM), glucose-6-phosphate (G-6-P) (0.86–30 mg mL^−1^), dehydrogenase (0.4–40 U mL^−1^), and magnesium ions (3.3–10 mM).

The effect of the concentration of HLM on the enzymatic reaction process was tested. The concentration of metabolites increased in the HLM concentration range 0.25–1.0 mg mL^−1^, while the concentration and number of metabolites formed did not increase above an HLM concentration of 1 mg mL^−1^. The concentration of oligonucleotides in the incubated samples was found to be an important parameter. Below 30 μM, no metabolites were observed, which is probably due to the fact that not all of the active sites of the enzyme were saturated with oligonucleotides. On the other hand, it may have been that metabolites were formed, but their concentrations were lower than the detection limit of the developed method. The extent to which dehydrogenase concentration affected the metabolism of the biomolecules studied was also tested. The results indicated that neither very low (0.4 U mL^−1^) nor high (40 U mL^−1^) concentrations yielded satisfactory results. The influence of the concentration of NADP was also tested. It was observed that increasing this concentration in the range 1–3 mM increased the concentration of metabolic products. However, no further increase in the number or concentration of metabolites was noticed above 3 mM.

Based on the results, the following parameter values were selected for subsequent studies: 12 h of incubation time, 1 mg mL^−1^ HLM, 30 μM ISIA18 and ISI20, 3 mM NADP/NADPH, 20 mg mL^−1^ G-6-P, 20 U mL^−1^ dehydrogenase, and 10 mM MgCl_2_.

### Identification of ASO metabolites

After selecting the optimal sample preparation method and optimizing the incubation conditions, proper incubation of the phosphorothioate oligonucleotides ISIA18 and ISI20 was performed.

**Fig. 4a–b:**
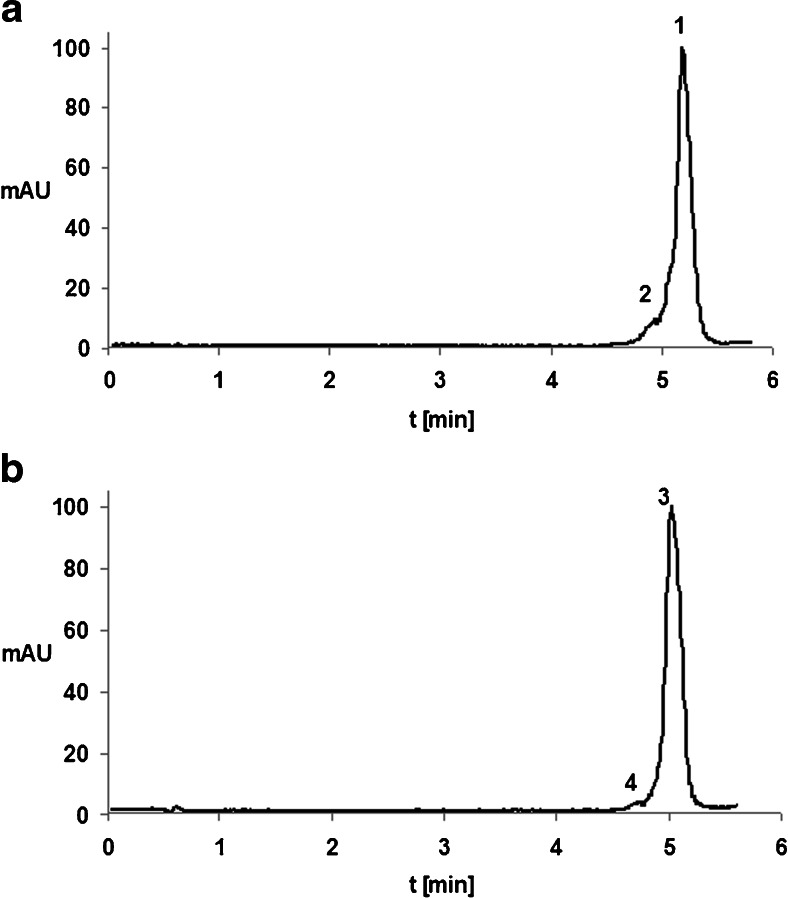
Total ion chromatograms of the products of the incubation of oligonucleotides with HLM for 12 h **a** ISI20, **b** ISIA18. Analytical conditions: chromatographic column, Hypersil Gold C18; mobile-phase composition, 150 mM HFIP/5 mM DMBA and MeOH; gradient elution from 20 % v/v to 45 % v/v MeOH in 10 min; column temperature, 50 °C; autosampler temperature, 40 °C; flow rate, 0.3 ml/min; drying/shielding gas temperature, 350/400 °C; nebulizer gas pressure, 20 psi; skimmer voltage, 60 V, octopole voltage, 750 V; capillary voltage, 4000 V; fragmentor voltage, 150 V. Notation: *1* ISI20, *2* ISI19, *3* ISIA18, *4* ISIA17

IP RP UHPLC Q-TOF MS analysis of samples was performed after incubating the oligonucleotides with enzymes in the microsomal fraction. The sensitivity of the method was 1.2–1.5 μg mL^−1^ based on the limit of quantification. Figure 4 presents TIC chromatograms from the analysis of samples incubated for a period of 12 hours. The results show that only one metabolite was created, which was one nucleotide shorter at the 3′ end. Of course, it is possible that more metabolites were formed but that their concentrations were below the detection limit. This small number of metabolite signals may also be due to ion suppression by other compounds and interferences in the samples. The low degree of metabolism in the HLM system may also be a result of the nature of the test compounds—ASOs are thought to be highly resistant to enzymes.

**Fig. 5a–c:**
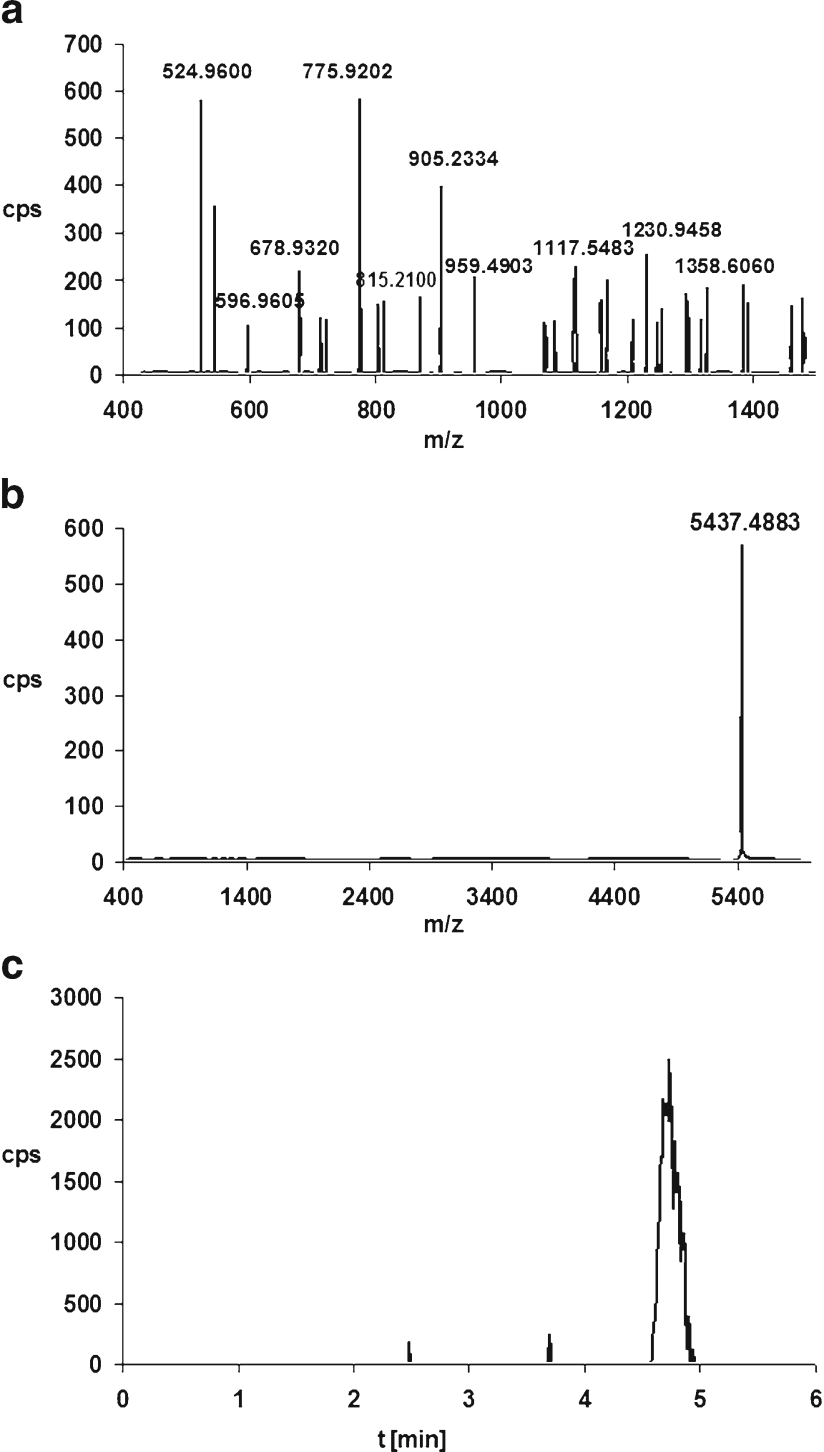
Scheme for identifying the metabolites of ISIA17 in the sample incubated with HLM (see also Fig. 4b). **a** Full-scan mass spectrum of ISIA17. **b** Mass spectrum after deconvolution. **c** Extracted ion chromatogram for the ion at *m*/*z* 775.920 Da

**Fig. 6a–c:**
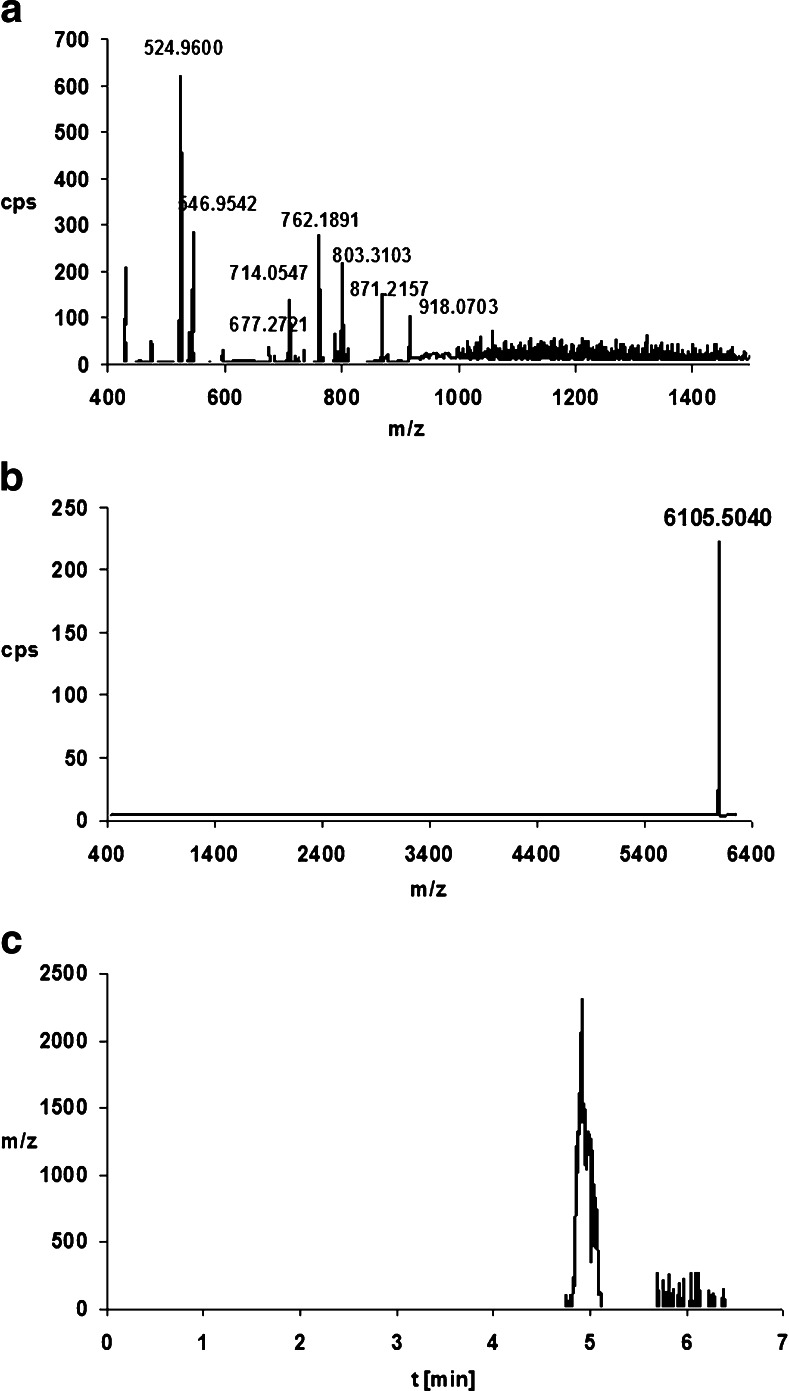
Scheme for identifying metabolites of ISI19 in the sample incubated with HLM (see also 4a). **a** Full-scan mass spectrum of ISI19. **b** Mass spectrum after deconvolution. **c** Extracted ion chromatogram for the ion at *m*/*z* 762.189 Da

The tested oligonucleotides and their metabolites were identified using the deconvolution method. The extracted ion chromatogram (EIC) for the selected and defined *m*/*z* values was also applied for this purpose. These values had previously been determined based on the mass spectra obtained for synthetic standards of oligonucleotides and their metabolites (Table 1). The ion with the greatest intensity was selected for both metabolites (i.e., *m*/*z* = 775.920 Da for ISIA17 and *m*/*z* = 762.189 Da for ISI19). The application of both methods to the determination of metabolite structure together with the full-scan spectra allowed us to confirm the presence of metabolites of ISIA18 and ISI20. Figures 5 and 6 present the full-scan spectra for these ASO metabolites (see also Fig. 4), together with the deconvolution and EIC results.

We examined the dependence of ASO enzymatic degradation on the incubation time using HLM. The results demonstrated that no metabolite was formed during the first four hours of incubation, although a metabolite was present in the sample after 12 hours. However, after a further 24 hours of incubation, neither the previously observed metabolite nor any other products of metabolism were observed. The enzymatic reactions of the two compounds ISIA18 and ISI20 were also compared. The results indicated that ISI20 is more resistant to degradation than ISIA18; this is because ISI20 has more nucleotides in its sequence than ISIA18. The metabolite of ISIA18 was detected after 8 hours, while that of ISI20 appeared after 12 hours.

In addition to ISIA18 and ISI20, the 17-mer and 19-mer synthetic metabolites ISIA17 and ISI19 were also incubated with HLM. Each resulting metabolite (16-mer ISIA16 and 18-mer ISI18, respectively) was one nucleotide shorter than its parent compound (ISIA17 and ISI19). This proves that, regardless of the length of the polynucleotide chain, metabolites are produced by the same mechanism.

## Conclusions

UHPLC was shown in this work to be a very effective analytical tool for the separation of ASOs and their metabolites. Its advantages include short analyses, high resolution, as well as reduced solvent and sample usage. The latter is a crucial benefit not only in relation to cost but also (especially) in the studies of biological samples. The mixture of HFIP, DMBA, and methanol was found to be the optimal mobile phase since it enables complete separation as well as a higher MS detection sensitivity than when TEA or HA is used. Mobile phases consisting of TEA, HFIP, and methanol are commonly used in oligonucleotide studies, but the contamination of mass spectrometers with TEA poses a significant problem (due to the memory effect of TEA). Consequently, DMBA proved to be an improvement on TEA. The fragmentation of phosphorothioate oligonucleotides was studied using MS/MS in order to apply this technique to the identification of metabolites. However, MS/MS was ultimately not used for this purpose as the product ion spectrum was the same regardless of the compound tested. Consequently, the identification of metabolites was achieved using deconvolution (accurate molecular masses) and EIC. Choosing the best method of preparing the studied compounds proved to be a complex task, as these compounds have a high affinity for proteins. However, it was found that simple liquid–liquid extraction using phenol/chloroform can be applied. The selected method enabled the purification and isolation of ASOs from enzymatic samples with a recovery rate of 95 %. In vitro metabolism studies using human liver microsomes demonstrated that, regardless of the length of the polynucleotide chain, the metabolites were produced by the same mechanism: enzymatic cleavage at the 3′ end of the sequence. Moreover, this work has shown, for the first time, that phosphorothioate oligonucleotides with long polynucleotide chains are more resistant to enzymatic degradation than phosphorothioate oligonucleotides with short polynucleotide chains. We can therefore conclude that human liver microsomes with enzymatic activity could be very useful for studying the metabolism of phosphorothioate oligonucleotides.

## Abbreviations

ASO: Antisense oligonucleotides
DMBA: *N*,*N*-Dimethylbutylamine
ESI: Electrospray ionization
HA: Hexylamine
HFIP: 1,1,1,3,3,3-Hexafluoro-2-propanol
HLM: Human liver microsomes
IP RP HPLC: Ion-pair reversed-phase high-performance liquid chromatography
MS: Mass spectrometry
Q-TOF MS: Quadrupole time-of-flight mass spectrometer
SPE: Solid-phase extraction
TEA: Triethylamine
UHPLC: Ultra high performance liquid chromatography
